# Phenotypic and genetic stepwise changes in *Staphylococcus aureus* during *in vitro* adaptive laboratory evolution under the selective pressure of tigecycline

**DOI:** 10.1128/aac.00072-25

**Published:** 2025-03-26

**Authors:** Honghao Huang, Peng Wan, Yiyi Chen, Xinyue Luo, Yizhen Zhu, Wanxin Lin, Yan Chen, Zhenling Zeng

**Affiliations:** 1 National Risk Assessment Laboratory for Antimicrobial Resistance of Animal Original Bacteria, South China Agricultural University 12526, Guangzhou, Guangdong, China; 2 Guangdong Provincial Key Laboratory of Veterinary Pharmaceutics Development and Safety Evaluation, South China Agricultural University 12526, Guangzhou, Guangdong, China; 3 Department of Infectious Diseases, Sir Run Run Shaw Hospital, Zhejiang University School of Medicine 56660, Hangzhou, Zhejiang, China; The Peter Doherty Institute for Infection and Immunity, Melbourne, Victoria, Australia

**Keywords:** adaptive laboratory evolution, *in vitro *selection, *Staphylococcus aureus*, tigecycline, *yycH *gene

## Abstract

Compared with tigecycline-resistant gram-negative bacteria, tigecycline adaptive laboratory evolution (ALE) has been preliminarily performed in *Staphylococcus aureus*. This study aims to develop higher-level tigecycline-resistant *S. aureus* mutants (TRSAms) and explore the mechanisms behind decreasing susceptibility to tigecycline. In this study, *S. aureus* strains were cultured in serial-increasing concentrations of tigecycline and successfully obtained high-level TRSAms. Different phenotypic changes in high-level TRSAms were assessed by growth rate measurement, autolysis assays, mutant frequency determination, and virulence evaluations *in vivo* and *in vitro*. The phenotypes of fitness cost showed significant differences in these high-level TRSAms. Whole-genome sequencing analysis detected synchronous mutations between *yycH* and *fakA* repeatedly in three high-level TRSAms from different parent strains. Further cloning experiments demonstrated that the complementary *yycH* gene increased susceptibility to tigecycline in TRSAms, and deletion mutant construction and complementation of Glu283Ter YycH confirm its critical role in tigecycline susceptibility in *S. aureus*. We also scanned the global genome to evaluate clinical importance; mutations on *rpsJ* detected in this study are associated with the MRSA ST5-t002 isolates and omadacycline selective mutants. In summary, we described a complete trajectory of phenotypic and genotypic changes in the ALE process for decreasing susceptibility to tigecycline in *S. aureus*. It is considered that the *yycH* gene has been involved in decreasing tigecycline susceptibility in *S. aureus*.

## INTRODUCTION

*Staphylococcus aureus*, a bacterial pathogen, causes infectious diseases such as pneumonia, endocarditis, and bacteremia (1). Tigecycline is a type of glycylcycline antimicrobial agent that is effective against multidrug-resistant bacteria (2). However, tigecycline-resistant *S. aureus* (TRSA) strains have recently been identified in animal and human sources (3–5). It is urgent to clarify the molecular mechanisms of tigecycline-resistant *S. aureus* to prevent and control the spread of TRSA.

Adaptive laboratory evolution (ALE) is a research tool that can be used to investigate the evolution of decreasing susceptibility and discover prospective therapeutic targets (6). For instance, a single mutation on *farR* was detected in the rhodomyrtone-resistant *S. aureus* mutant (7). Further molecular research confirmed that the actual target of rhodomyrtone is the cytoplasmic membrane regulated by *farR* (8). Similar processes were performed under tigecycline pressure in *Escherichia coli* and *Acinetobacter baumannii*. The *mlaA* gene, which belongs to the adenosine-triphosphate binding cassette transport system and can maintain outer membrane lipid asymmetry, is involved in conferring resistance to tigecycline in *E. coli* (9). The *trm* gene encoding an S-adenosyl-L-methionine-dependent methyltransferase was mutated to decrease susceptibility to tigecycline in *A. baumannii* (10). Discovered in genetic stepwise changes, these mechanisms expanded the resistant mechanism in clinical tigecycline-resistant isolates. In *S. aureus*, ALE studies were performed under the selective pressure of tigecycline, and tautological mutations were found to occur in *rpsJ* and *mepA* genes (11–13). Nonetheless, the MICs of tigecycline for selective mutants observed in these ALE studies were found to be less than 16 mg/L. This concentration is comparatively lower than that reported for other bacterial species, which may explain why the additional resistance mechanisms have not been identified in *S. aureus*.

This study aimed to unravel phenotypic and genetic comprehensive changes in TRSA mutants (TRSAms) associated with tigecycline resistance and explore the potential mechanism for maintaining tigecycline susceptibility in *S. aureus*.

## MATERIALS AND METHODS

### Selection of decreasing susceptibility to tigecycline

As previously described (14), three strains of *S. aureus*, ATCC 25923, ATCC 29213, and ATCC 43300, were cultivated by serial passage under the selective pressure of tigecycline. Tigecycline-resistant mutants were cultured in tigecycline-free agar with 10 passages to confirm the stability of mutants. Tigecycline-resistant mutants were named by the abbreviations of strains and altered minimum inhibitory concentration (MIC) against tigecycline. The selection assays were concluded when the MICs of tigecycline ceased to increase for 10 consecutive days.

### Antimicrobial susceptibility testing

*S. aureus* ATCC 29213 was set to be the quality control strain. The MIC of tigecycline was determined using the broth microdilution method following the European Committee on Antimicrobial Susceptibility Testing (version 12.0) (15). Other antimicrobial agents were tested using the agar microdilution method as per the guidelines of the Clinical and Laboratory Standards Institute (16).

### Efflux pump inhibition assay

Efflux inhibition assays were performed to explore the association between efflux pump activity and tigecycline resistance. The broth microdilution method was adopted to determine the MICs against tigecycline in the presence or absence of efflux pump inhibitors (EPIs) (Yuanye, Shanghai, China). The EPIs used in this study are as follows: 2 µM carbonyl cyanide 3-chlorophenylhydrazone (CCCP), 100 mg/L 1-(1-naphthylmethyl)-piperazine (NMP), and 20 mg/L reserpine (RS). The reduction in inhibition levels was evaluated using twofold dilution (TFD) in the assays.

### RNA extraction and transcript level evaluation

The gene expression levels of *mepA* were evaluated by RT-qPCR. Total RNA was extracted using an RNA isolation kit (Vazyme, Nanjing, China) and immediately reverse transcribed using a cDNA synthesis kit (Vazyme, Nanjing, China). qPCR was performed using a Two-Step SYBR Green premix (Takara, Shiga, Japan) in the CFX Connect (Bio-rad, Hercules, USA) with the primers listed in Table S1. The *gyrB* gene was selected as the housekeeper. Cq values were converted to quantity values, and the ΔΔCT method (relative) was used to calculate the relative transcript levels of *mepA* and *yycH*.

### Fitness cost evaluation

Fitness cost was evaluated by growth rate measurement, autolysis assays, and mutant frequency determination.

Growth rates were measured as follows. Strains were grown overnight and then incubated (dilution 1/100) in broth at 37°C, 180 rpm. The optical density at 600 nm (OD_600_) was used to evaluate the density of bacterial cells and determined every 30 min for 8 h. Growth rates were statistically compared through slope analysis by linear regression.

Autolysis assays were performed as follows. Strains were grown overnight and then washed twice in phosphate-buffered saline (PBS) before being resuspended in PBS. OD_600_ was adjusted to about 1.00. Lysostaphin (0.5 mg/L) (Sangon, Shanghai, China) was supplemented to induce autolysis. The OD_600_ values of all strains were read every 30 min for 4 h. The initial value was set as 100%, and all the other values were calculated as percentages of the initial data.

Mutant frequencies were determined as follows. Strains were grown overnight and then resuspended in 1/10 volume normal saline. Continuous dilution was performed eight times in 10-fold using normal saline. The colonies were spread onto the brain heart infusion (BHI) agar plates supplemented with and without rifampicin (100 mg/L), and the numbers were counted after 24 h. Mutant frequencies were calculated by dividing the number of rifampicin-resistant mutants by the total number of cells.

### Virulence evaluation

The hemolytic activity *in vitro* was evaluated. Strains were grown overnight and washed with normal saline. The OD_600_ of cells was adjusted to 1.0. Sterilized sheep blood diluted with PBS buffer was used to make 4% red blood cells (RBCs). Bacterial cells and 4% RBCs were mixed in a 1:1 ratio. The mixtures were centrifuged at 1,500 × *g* for 5 min after 3-h incubation at 37°C. The OD_600_ values of supernatants were used to evaluate hemolytic activity. The equal volume of PBS buffer was set as the negative control. The results were calculated by subtracting the values of the negative control.

An *in vivo* model of *S. aureus* infection was structured in *Galleria mellonella*. Different TRSAm strains, including MICs of tigecycline from 2 to 128 mg/L, and their parent strains were cultured to 10^9^ colony-forming units (CFUs)/mL. Cultured bacterial cells were centrifuged and suspended in normal saline. *G. mellonella* was injected with 10 µL bacterial cell suspension or normal saline for infection or control, respectively. The survival situations of *G. mellonella* were observed every 8 h for 24 h.

### Whole-genome sequencing and data analysis

Bacterial DNA was extracted with a kit according to the manufacturer’s standard protocol. The Illumina Novaseq 6000 sequencing platform (Illumina, San Diego, USA) was utilized for whole genome sequencing (WGS). CLC Genomics Workbench version 10.1 (Qiagen, Hilden, Germany) was used for assembling Illumina Novaseq sequences. For scanning global genomic data, an own database was made by ABRicate (https://github.com/tseemann/abricate). The selected genome data were downloaded from the GenBank database (Table S3).

Prokka version 1.14.6 was applied to annotate the WGS data of the strains (17). Single nucleotide variants (SNVs) were analyzed by Snippy version 4.6.0 (https://github.com/tseemann/snippy). The detected SNVs were amplified using special primers (Table S1) and Sanger sequencing to confirm nucleotide sequences. The molecular types of *S. aureus* were identified by mlst (https://github.com/tseemann/mlst) and spaTyper (https://github.com/mjsull/spa_typing). To infer a phylogeny analysis, core-genome alignment was constructed by Parsnp (18) with random reference. A maximum-likelihood tree of the filtered core genomes was inferred with a GTRGAMMA replacement model using RaxML (19).

The produced WGS data were deposited in the GenBank database under the BioProject accession number PRJNA1066721.

### Competent cells’ preparation and electroporation

*S. aureus* cells were grown overnight in BHI broth, and 1 mL of overnight culture was added to 100 mL of fresh BHI. After the cells were grown at 37°C, the samples were centrifuged at 180 rpm until the OD_600_ values were 0.2. Then, the cells were washed with ice-cold 0.5 M sucrose aqueous solution and resuspended in 1 mL of 0.5 M sucrose aqueous solution.

Approximately 100 µL of cells suspended in sucrose aqueous solution was added to 1 µg of DNA and incubated on ice for 15 min. Electroporation was performed using the MicroPulser electroporator (Bio-Rad, Hercules, USA) with the following parameters: 2.5 kV, 200 Ohm, and 25 µF. Electroporated cells were revived in tryptose soya broth (TSB) for 2 h, then centrifuged at 5,000 × *g*, resuspended in 100 µL of TSB, and incubated in antimicrobial selective tryptose soya agar (TSA) plates. PCR amplification and Sanger sequencing were performed using primers vLI-F and vLI-R to confirm electroporation successfully.

### Functional complementation of mutants

Complementary plasmids were constructed using shuttle vector pLI50, which was performed as described previously (20). In brief, the fragments were amplified and purified using primers (Table S1). Target genes and their original promoters were amplified from the genomic DNA of parent strains. Finally, the genes and promoters were inserted into the vector via a seamless cloning kit (Tsingke, Beijing, China). The point mutations Asp60Tyr and Lys57Gln on the *rpsJ* gene were conducted with the site-directed mutagenesis kit (Sangon, Shanghai, China), and the required primers used for mutagenesis were designed using PrimerX (https://www.bioinformatics.org/primerx/index.htm). All plasmids were extracted using the manufacturer’s kit (Tiangen, Beijing, China). The plasmids were electrotransformed into *S. aureus* RN4220 and subsequently into TRSAms. All plasmids and strains constructed in this study are listed in Table 1.

**TABLE 1:** Strains and plasmids used in the study

	Descriptions	Source
*E. coli* strains		
DH5α	Recipient strains of construction of plasmids	Tsingke, Beijing, China
*S. aureus* strains		
ATCC 25923	*S. aureus* standard strain	Laboratory collection
ATCC 29213	*S. aureus* quality control strain	Laboratory collection
ATCC 43300	MRSA standard strain	Laboratory collection
RN4220	Engineering *S. aureus* strain	Laboratory collection
29213Δ*yycH*	*yycH* gene deletion mutant of ATCC 29213	This study
Plasmids		
pLI50	Expression vector in *S. aureus* with single copy	(20)
pLI50-yycH	pLI50 carrying the *yycH* gene and original promoter	This study
pLI50-myycH	pLI50 carrying the mutant *yycH* gene and original promoter	This study
pLI50-fakA	pLI50 carrying the *fakA* gene and original promoter	This study
pLI50-rpsJ	pLI50 carrying the *rpsJ* gene and original promoter	This study
pLI50-rpsJ_K57Q_	Point mutation K57Q based on pLI50-S10	This study
pLI50-rpsJ_D60Y_	Point mutation D60Y based on pLI50-S10	This study
pLI50-rpsJ_K57Q +D60Y_	Point mutations K57Q and D60Y based on pLI50-S10	This study
pHoss-1	Shuttle vector in *E. coli* and gram-positive bacteria	(21)
pHoss-hyycH	pHoss-1 carrying the flanked homologous sequences of the *yycH* gene	This study

### Deletion mutant construction and complementation

To investigate the function of *yycH* alone, the *S. aureus* ATCC 29213 was selected for the construction of a deletion mutant and its complementation.

The shuttle vector pHoss-1 (21) was employed to delete *yycH* through allelic exchange. In brief, more than 800 bp of DNA regions flanking the *yycH* gene were amplified and purified using a commercial kit (Tiangen, Beijing, China) and subsequently cloned into pHoss-1 linearized by *Eco*R I (NEB, England) using a seamless cloning kit (Tsingke, Guangzhou, China). The resulting deletion mutant plasmid, pHoss-hyycH, was then electrotransformed into ATCC 29213 as described above. Electrotransformants were cultured on TSA plates supplemented with erythromycin (15 mg/L) at 42°C for 24 h, and this process was repeated twice. Subsequently, the colonies grown in erythromycin TSA were incubated in TSB without antibiotics at 30°C for 24 h, also repeated twice. Finally, cells were selected by spreading on TSA plates containing 1.5 mg/L Anhydrotetracycline Hydrochloride (Macklin, Shanghai, China) and incubating plates at 37°C for 24 h. Colonies that were susceptible to erythromycin were amplified and subjected to Sanger Sequencing to confirm the deletion of *yycH.* The deletion mutant strain was designated 29213Δ*yycH*.

To perform the complementation, the original *yycH* was amplified from ATCC 29213, while the mutant *yycH* was amplified from m29213T128 and then cloned into pLI50 as described above. Complementation plasmids pLI50-yycH and pLI50-myycH were then electrotransformed into 29213Δ*yycH*.

## RESULTS

### Antimicrobial resistance of *in vitro* selective mutants

**Fig 1:**
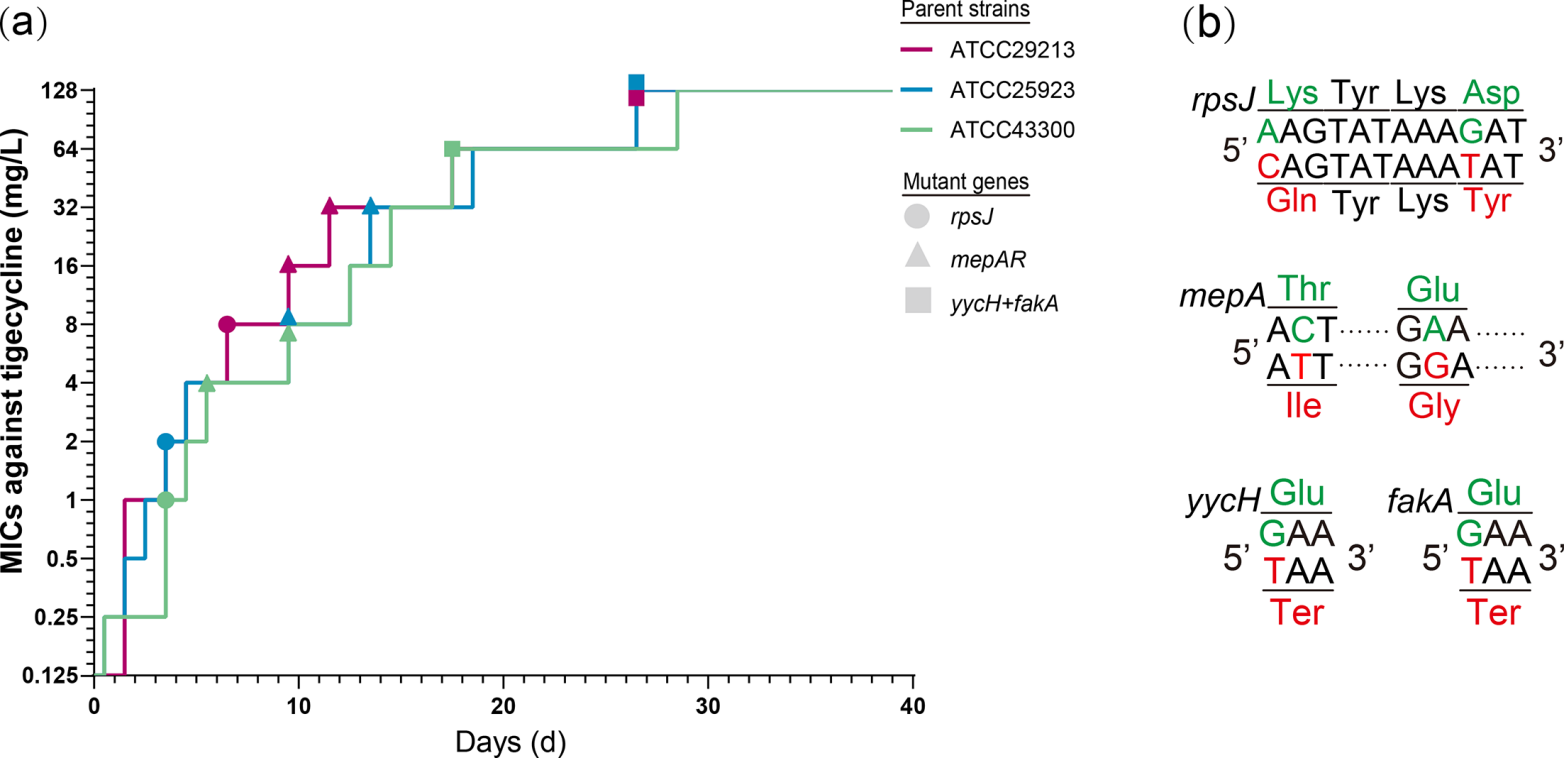
Development of tigecycline resistance in ALE. (a) The tigecycline resistance of three *S*. *aureus* standard strains was developed in ALE. Various icons represent linked mutant genes, and their colors were filled with line colors representing parent strains. (b) Mutations were detected in the genes. Green letters indicate the original sequences of nucleotides and amino acids in parent strains, while red ones indicate the substitution mutations causing nucleotides and amino acids.

The MICs of tigecycline in ATCC 25923, ATCC 29213, and ATCC 43300, three *S*. *aureus* strains, increased to 16 mg/L within 16 days. The letter T was added to the abbreviations of mutant strains with changed MICs in the process of *in vitro* selection (for instance, m25923T1 meant that the MIC of tigecycline in ATCC 25923 rose to 1 mg/L). After 10 days of bottleneck, the MICs of tigecycline increased to 64 and 128 mg/L (Fig. 1a). To describe the results of the following assays, the strains with an MIC of tigecycline ≤16 mg/L were designated as low-level resistant strains, and the strains with an MIC of tigecycline more than 16 mg/L were regarded as high-level TRSAms to distinguish the mutants selected in previous similar ALE studies.

All TRSAms with TGC MIC ≥ 32 mg/L were collaterally resistant to the other earlier tetracyclines (Table 2), and the resistance level of tetracyclines was similar to that of tigecycline. Interestingly, collateral susceptibility in β-lactam was detected in the mutants of MRSA ATCC 43300 (Table S2).

**TABLE 2:** MICs of parental and mutant strains

	MICs (mg/L) of^*a*^						
Strains	TET	DOX	MNO	TGC	CCCP^*b*^	NMP^*b*^	RS^*b*^
ATCC 29213	0.5	0.125	0.125	0.125	0.06	0.125	0.125
m29213T2	2	0.25	0.25	2	0.25	1	0.25
m29213T8	8	1	0.25	8	0.25	0.5	4
m29213T32	16	4	2	32	4	4	8
m29213T128	32	8	8	128	32	32	32
ATCC 25923	0.5	0.125	0.125	0.125	0.06	0.125	0.125
m25923T2	4	1	0.5	2	0.25	0.25	0.25
m25923T8	8	2	2	8	0.5	1	2
m25923T32	16	4	4	32	2	2	8
m25923T128	32	16	8	128	32	32	64
ATCC 43300	0.5	0.125	0.125	0.125	0.125	0.125	0.125
m43300T2	2	0.5	0.5	2	0.125	0.25	0.25
m43300T8	8	2	1	8	0.5	1	4
m43300T32	16	4	4	32	4	4	16
m43300T128	32	8	8	128	32	32	64

^*a*^ TET, tetracycline; TGC, tigecycline; DOX, doxycycline; and MNO, minocycline.

^*b*^ Determination of MICs against tigecycline with the broth supplemented with CCCP (2 mM), NMP (100 mg/L), or RS (20 mg/L).

### Expression of mepA increased with the development of tigecycline resistance

**Fig 2:**
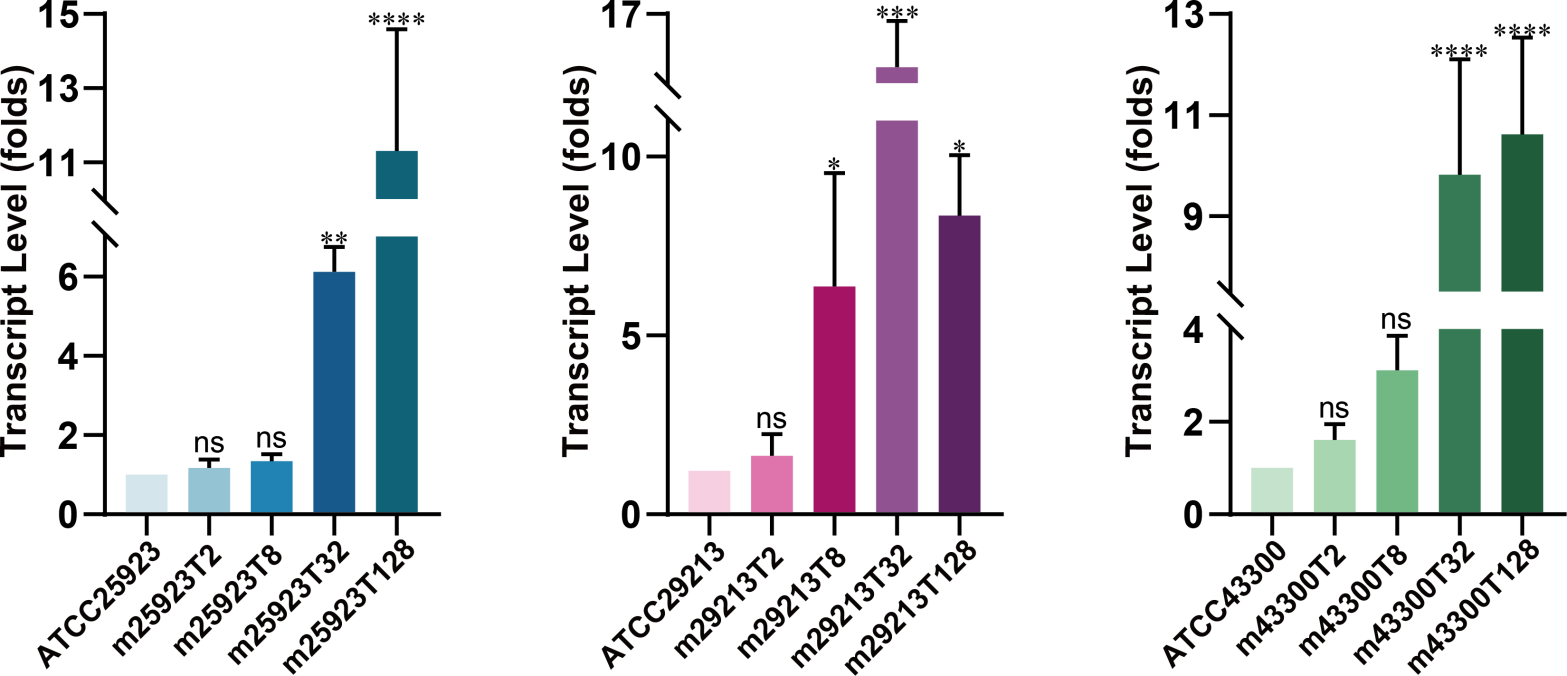
The transcript levels of the *mepA* gene in tigecycline-resistant *S. aureus* mutants. The gene expressions were determined by RT-qPCR. Data are mean with SD shown as an error bar. Statistical significance was determined by one-way analysis of variance (ns, no significance, *P* ≥ 0.05; *P < 0.05; **P < 0.01; ***P < 0.001; and ****P < 0.0001).

The transcript levels of efflux pump gene *mepA* were determined by RT-qPCR. The *mepA* genes of three lineages of *S. aureus* were consistently overexpressed in mutants with MIC against tigecycline over 32 mg/L (Fig. 2). In the presence of EPIs, the MICs against tigecycline were reduced by 2–16 TFD (Table 2). The efflux inhibition of RS was weaker than that of CCCP and NMP. Taken together, the statistically significant overexpression of the *mepA* gene confers tigecycline resistance in the ALE.

### Fitness cost in tigecycline-resistant mutants

To describe the phenotypic trajectory of TRSAms, five strains from each serial parent strain were selected to evaluate fitness costs, and their MICs against tigecycline were progressively increased by fourfold increments.

**Fig 3:**
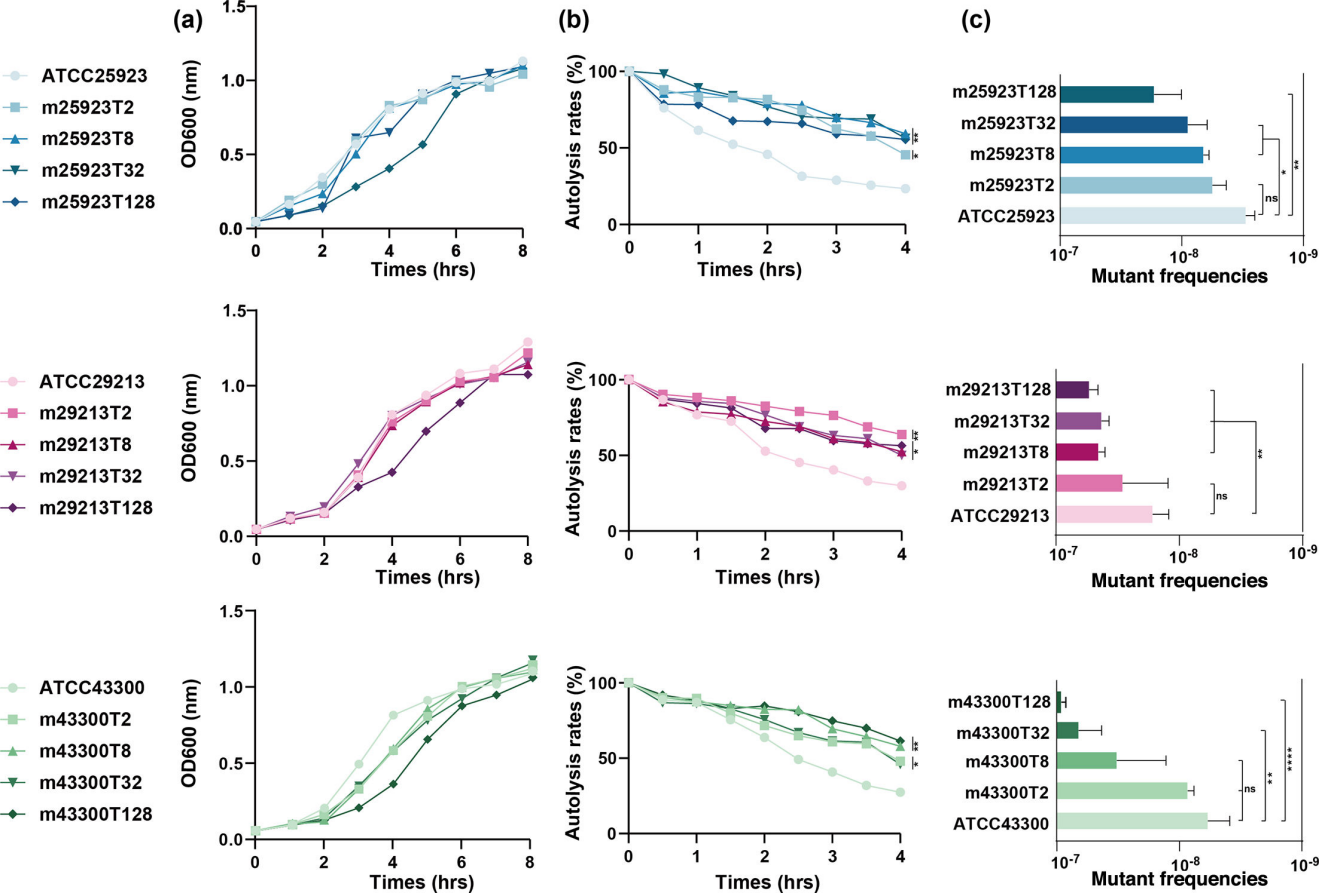
Fitness cost in tigecycline-resistant *S. aureus* mutants. The changes in growth rate, autolysis, and mutant frequency were described. Blue, red, and green tone graphs indicate *S. aureus* lineages ATCC 25923, ATCC 29213, and ATCC 43300, respectively. The depth of colors represents the level of tigecycline resistance (the darker the color is, the higher the MIC value of tigecycline will be). (a) Growth curves were based on optical density, and each point was a recorded measurement. (b) Autolysis activity was estimated using percentages of the optical density of initial data. (c) The spontaneous mutant frequencies of strains were determined under the selective pressure of rifampin. All the data are mean with SD (error bar in panel c, absent in panels a and b) of three independent assays. Statistical significance was determined by one-way analysis of variance (ns, no significance, *P* ≥ 0.05; *P < 0.05; **P < 0.01; ***P < 0.001; and ****P < 0.0001).

Low-level TRSAms were not statistically different from parent strains except for the lineages of ATCC 43300, and all the high-level TRSAms (MIC = 128 mg/L) measured showed slower growth rates than the others (Fig. 3a). All the TRSAms exhibited significantly stronger autolysis activity than parent stains, and no significant differences were observed among themselves (Fig. 3b). Mutant frequencies were increased 10-fold in highest-level TRSAms than parent strains (Fig. 3c).

Overall, TRSAms exhibited slower growth limited in high-level resistance. Weakened autolysis activity was detected in all TRSAms, and no difference was found between low- and high-level strains. Different lineages of *S. aureus* strains reduced genomic stability while decreasing susceptibility to tigecycline.

### Attenuation of virulence in tigecycline-resistant mutants

**Fig 4:**
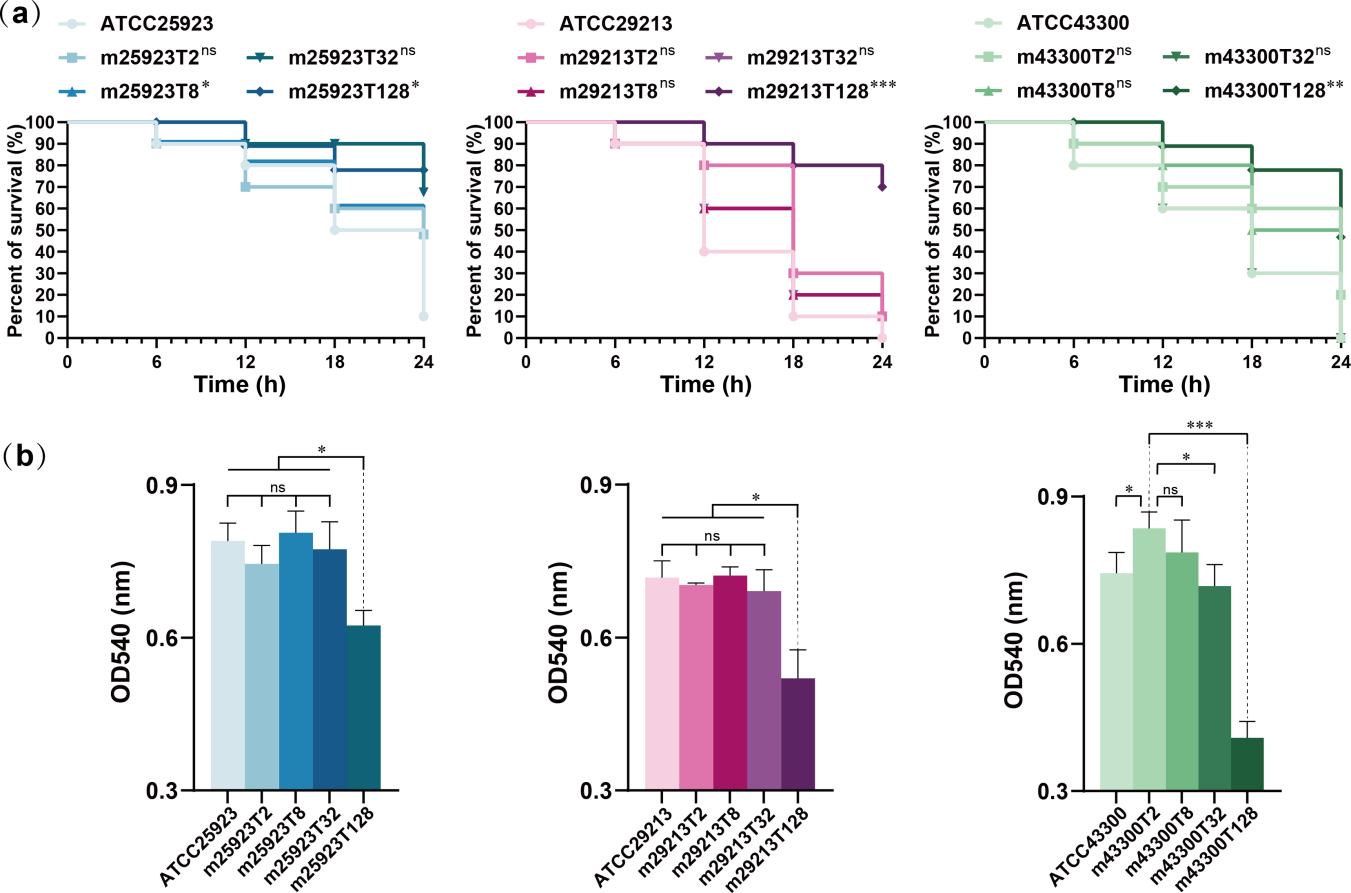
Virulence changes in tigecycline-resistant *S. aureus* mutants were evaluated *in vitro* and *in vivo*. (a) Survival curve of infected *Galleria mellonella*. The statistical significance was determined by the log-rank (Mantel-Cox) test (ns, no significance, *P* ≥ 0.05; *P < 0.05; and ***P < 0.001). (b) Hemolytic activity of strains. Data are mean with SD shown as an error bar, and statistical significance was determined by one-way analysis of variance (ns, no significance, *P* ≥ 0.05; *P < 0.05; and ***P < 0.001).

The hemolytic activity of high-level TRSAm strains (MIC = 128 mg/L) showed statistically significant decreases to that of low-level strains (Fig. 4b). The *in vivo* infection model once again revealed the phenomenon that the survival of *G. mellonella* was significantly higher in high-level TRSAms (Fig. 4a). When infection with parent strains resulted in mortality, more than half of the high-level groups survived.

### Genetic stepwise changes in *in vitro* selective mutants

To explore the resistance mechanism in high-level TRSAms, WGS analysis was performed on three higher-level TRSAm strains, and Sanger sequencing was used to confirm the nucleotide sequences of the involved genes in all mutants.

High-level TRSAms were analyzed by WGS analysis. The synchronous mutations in *yycH* and *fakA* genes were detected simultaneously in three lineages of parent strains (Fig. 1b). It was confirmed that these kinds of mutations were exclusively detected in high-level TRSAms rather than other strains. Amino acid mutations resulted from the same nucleotide substitution, specifically G > T. That is, glutamate turned into a termination codon. Glu242Ter in FakA was detected, and Met243 may be replaced as the initiation codon. In addition, Glu283Ter in YycH led to the deletion of eight amino acids (Glu283_Lys290del). Meanwhile, mutations in the *mepA* and *rpsJ* genes were identified sequentially across three lineages of parent strains. The *rpsJ* gene exhibited synchronous mutations, specifically Lys57Gln and Asp60Tyr. In the *mepA* gene, the mutations Thr29Ile and Glu287Gly were detected simultaneously in the ATCC 29213 and ATCC 25923 lineages, whereas only the Glu287Gly mutation was observed in the ATCC 43300 lineage.

### *yycH* but not *fakA* was involved in maintaining tigecycline susceptibility

These two genes were cloned into the shuttle vector pLI50 by the same protocol. pLI50-yycH and pLI50-fakA were acquired and electrotransferred into high-level TRSAms. The RT-qPCR was conducted to assess the transcript levels of yycH in the electrotransformants, revealing levels comparable to those of the parental strains (Fig. S1). The susceptibility of tigecycline was restored to 16–32 mg/L after the electrotransformation of pLI50-yycH to high-level TRSAm strains (Table 3). The efflux inhibitions detected in high-level TRSAms were still contained in the *yycH* complementary transformants. However, TRSAms electrotransformed with pLI50-fakA failed to have restored tigecycline susceptibility.

**TABLE 3:** MICs of deletion mutant and complementary strains

	MICs (mg/L) of^*a*^			
Strains	TET	DOX	MNO	TGC
m29213T128-pLI50-yycH	16	16	4	16
m25923T128-pLI50-yycH	16	8	4	32
m43300T128-pLI50-yycH	16	8	4	16
29213Δ*yycH*	0.5	0.125	0.125	0.125
29213Δ*yycH*-pLI50	0.5	0.125	0.125	0.125
29213Δ*yycH*-pLI50-yycH	0.5	0.125	0.125	0.125
29213Δ*yycH*-pLI50-myycH	64	8	4	2
RN4220	0.5	0.125	0.125	0.125
RN4220-pLI50-rpsJ	0.5	0.125	0.125	0.125
RN4220-pLI50-rpsJ_K57Q_	2	2	2	1
RN4220-pLI50-rpsJ_D60Y_	2	2	2	1
RN4220-pLI50-rpsJ_K57Q +D60Y_	8	4	4	2

^*a*^ TET, tetracycline; TGC, tigecycline; DOX, doxycycline; and MNO, minocycline.

To confirm the crucial role of *yycH* in decreasing susceptibility to tigecycline in *S. aureus*, the *yycH* gene was deleted in *S. aureus* ATCC 29213. However, the deletion mutant strain 29213Δ*yycH* did not exhibit a change in the MICs of tetracycline. In the complementation experiments, only the strain complemented with Glu283Ter YycH showed a significant increase in the MICs of tetracycline, which represents a 16-fold change (0.125–2 mg/L) Table 3.

### Clinical relevance of mutations in related genes

To confirm the resistance contribution of mutations in the *rpsJ* gene, the gene was cloned into pLI50 to create pLI50-rpsJ. Point mutation plasmids were named pLI50-rpsJ_K57Q_, pLI50-rpsJ_D60Y_, and pLI50-rpsJ_K57Q+D60Y_. *S. aureus* RN4220 electrotransformants carrying pLI50-rpsJ showed no change in MICs against tetracyclines. By contrast, the point mutation plasmids conferred tetracycline resistance in electrotransformants. The MICs of single-point mutations against tigecycline increased eightfold compared to *S. aureus* RN4220 (0.125–1 mg/L), and the existence of mutations K57Q + D60Y increased 16fold (0.125–2 mg/L).

**Fig 5:**
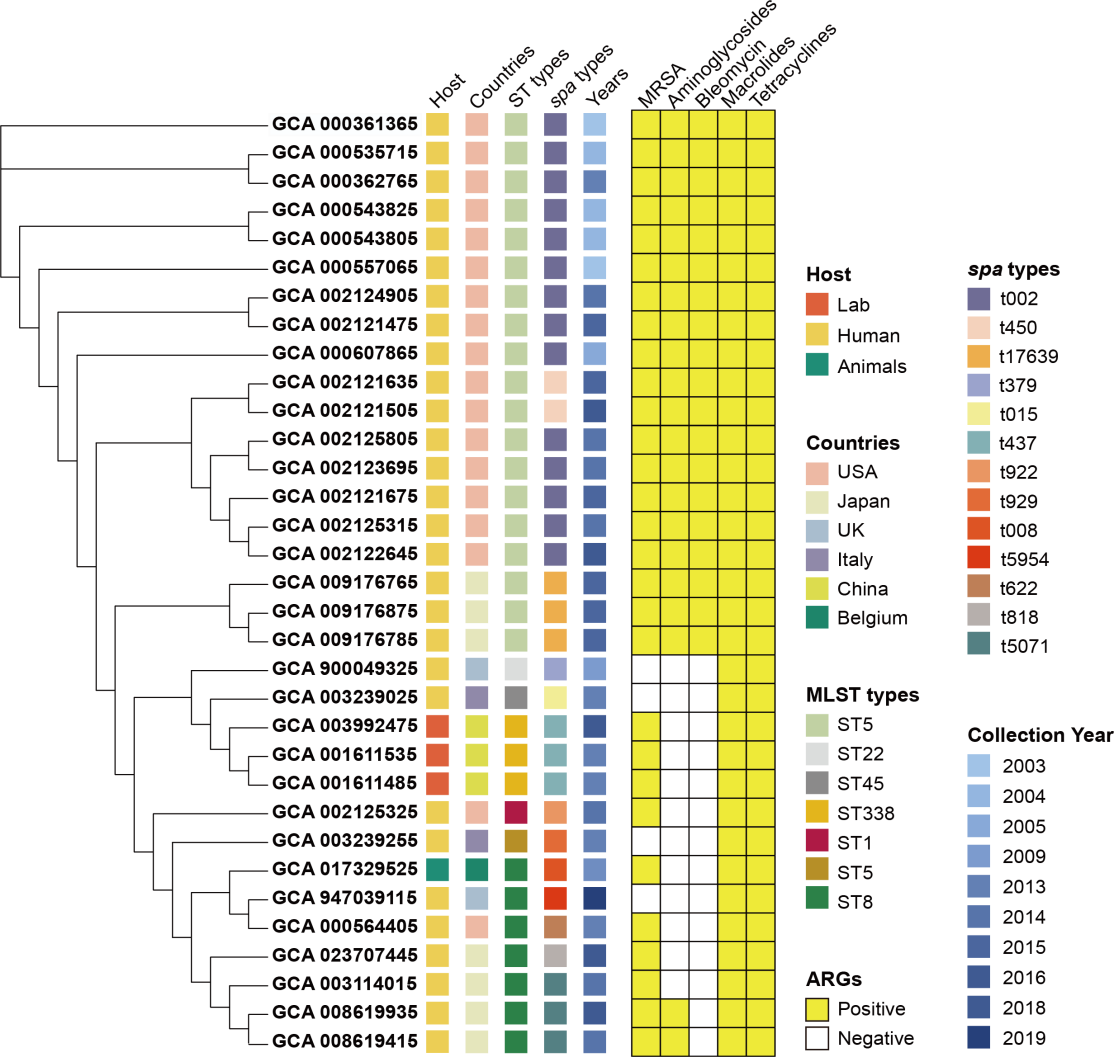
The phylogenetic tree of clinical isolates detected mutations on *rpsJ* that are the same as this study. A total of 33 *S*. *aureus* strains were detected with mutations Lys57Gln and Asp60Tyr in the *rpsJ* gene. The phylogenetic tree, constructed by maximum-likelihood method, contained strain information, molecular character, and antibiotic-resistant genes (classified as sorts of antibiotics).

To explore the clinical relevance of mutations detected in this study, we scanned global genomes. Results indicated that 33 genomes of humans, labs, and animals from different countries from 2003 to 2019 were found to have the same mutation on the *rpsJ* gene (Table S2). Phylogenetic analysis showed that mutations on *rpsJ* were related to the MRSA ST5-t002 prevalence in the USA from 2003 to 2016 (Fig. 5). The matched mutations on *mepA* or *yycH* identified in this study were not found in global genomes based on our investigation.

## DISCUSSION

To discover the universality of phenotypes and genotypes, three parent *S. aureus* strains were used to perform ALE in the present study. Even though the three parent strains utilized belong to separate evolutionary lineages, no variation in decreasing susceptibility to tigecycline was found during the ALE process.

The overexpression of *mepA* is demonstrated to be an important factor in tigecycline resistance in *S. aureus* (22). The transcript levels were significant in TRSAms. However, the transcript levels were not always increasing with tigecycline-resistant levels, which indicated that the selection of high-level TRSAms was not dependent on the overexpression of *mepA*. Correspondingly, efflux pump inhibition assays showed that efflux activity confers resistance to tigecycline in TRSAms. Notably, high-level TRSAms continue to demonstrate higher resistance than low-level ones, even when inhibited by EPIs. It also revealed that high-level TRSAm strains exhibited an unexplained resistance mechanism, with the exception of efflux activity.

The selective mutants with MICs of tigecycline greater than 16 mg/L were absent in previous studies (13). In this study, we acquired strains with higher resistance levels, and a more thorough adaptive laboratory evolutionary trajectory was provided by phenotypic evaluations, including fitness cost and virulence evaluation. Undoubtedly, the development of antimicrobial resistance is positively related to the fitness cost of host bacteria (23). However, the previous ALE study did not discover a significant fitness cost in the selective mutants similar to the low-level TRSAms in this study. In contrast, a significant fitness cost was detected in high-level TRSAm strains. It indicated that previous studies may be insufficient and accompanied by incomplete decreasing susceptibility. Like other gram-positive bacteria, *S. aureus* strains have specific cell wall structures associated with various antimicrobial resistances (24). Lysostaphin was used to induce autolysis in this study. The results showed that *S. aureus* cell walls were involved in the occurrence of tigecycline resistance rather than its further development. Mutant frequency was associated with genomic stability, and resistance to rifampicin was solely conferred by a genomic factor in *S. aureus*. Therefore, mutant frequencies were determined under the selective pressure of rifampicin. The results showed that the mutant frequencies of TRSAms increased as tigecycline susceptibility decreased.

Bacterial virulence usually changes with an increase in fitness cost. Virulence assessment was performed using hemolytic activity *in vitro* and the *G. mellonella* infection model *in vivo* (Fig. 3). The results suggest that strain virulence was inversely correlated with tigecycline resistance and exhibited a significant difference between low- and high-level TRSAms.

The phenotypic characteristics of high-level TRSAms determined in efflux inhibition and fitness cost evaluation differed from those of low-level TRSAms in this and other studies. It indicated that the novel unidentified tigecycline-resistant mechanism existed in high-level TRSAms.

To identify the exact mechanism, WGS and SNV analyses were performed between high-level TRSAm and parental strains. Two genes, including *fakA* and *yycH,* were detected in three lineage strains. The *fakA* gene encodes the fatty acid kinase FakA, which can incorporate extracellular fatty acids into the phospholipids of *S. aureus*. Glu242 of FakA was deleted, and the Glu283Ter mutation in YycH resulted in the deletion of eight amino acids. Glu242 of FakA is located on the central FakA-L in a protein domain study, and the authors reported that Glu242 is a conserved residue not involved in catalytic activity (25). The *walRK* (*yycFG*) is the only essential two-component system in *S. aureus* (26). As the auxiliary protein of WalRK, *yycH* plays an important role in the direct regulation of WalRK-dependent cell wall metabolism (27). The mutant *yycH* gene was confirmed to be involved in conferring resistance to vancomycin (28) and daptomycin (29) in *S. aureus*. Although insufficient evidence associated with *yycH* could be obtained, vancomycin susceptibility may be decreased in tigecycline *in vitro* selection (30). This study demonstrated that novel tigecycline-associated mutations occurred and accumulated in *rpsJ* and *mepAR* in low-level TRSAms (14). The synchronous mutations discovered between *yycH* and *fakA* genes were a significant discovery for a complete description of genetic changes in TRSAms.

However, the involvement of two genes in tigecycline resistance cannot be verified currently. To confirm whether synchronous mutant *yycH* and *fakA* genes are associated with tigecycline resistance, these two original genes were cloned into the vector separately and electrotransferred into high-level TRSAms. The results confirmed that only the *yycH* gene is involved in tigecycline resistance. Further deletion mutant construction and complementation experiments revealed that tigecycline susceptibility decreased if and only if the Glu283Ter mutation in YycH was present in *S. aureus*. A similar phenomenon was described in a previous study performed on ALE of *E. coli* under the selective pressure of tigecycline. The decrease in tigecycline susceptibility was not observed in *E. coli* ATCC 25922 with a deleted *mlaA* gene or its complementation with the original *mlaA* gene. However, the strains complementary with mutant *mlaA* conferred tigecycline resistance, with a fourfold increase in MICs (0.25–1 mg/L) (9).

To the best of our knowledge, this study is the first to report that the *yycH* gene is associated with tigecycline susceptibility in *S. aureus*. In previous reports, the mutant *yycH* was usually related to vancomycin susceptibility in *S. aureus* isolates (28). Vancomycin inhibits cell wall biosynthesis in *S. aureus*, and specific mutations in YycH reduce *walK* expression, leading to cell wall thickening and reduced vancomycin susceptibility (31). However, the Glu283Ter mutation on YycH detected in this research did not alter vancomycin susceptibility in high-level TRSAms. The yycH function may be preserved or compensated for through undefined mechanisms. Interestingly, the *yycH* gene was also involved in resistance to non-cell-wall-targeting antimicrobials. For instance, *yycH* deletions and insertions increase daptomycin resistance (29), even though the cell wall is not the primary target of daptomycin. This implies that YycH may play an unknown pleiotropic role in *S. aureus,* which may similarly underlie the decrease in tigecycline susceptibility mediated by the Glu283Ter YycH.

The canonical mechanisms of antimicrobial resistance can be summarized into five categories: target site alteration, efflux activity, drug inactivation, decreasing the uptake of drugs, and metabolism redirection (32). Current mechanisms of tigecycline resistance in *S. aureus* are limited to target site alteration and efflux activity. The first hypothesis that can be excluded is target-mediated resistance, given that the *yycH* deletion did not affect the MIC of tigecycline. Efflux activity was deemed unlikely because YycH lacks structural features characteristic of transporters, including transmembrane domains, and the MICs of tigecycline did not exhibit a persistent downward trend in efflux inhibition assays when efflux pump inhibitors were introduced. Drug inactivation is only documented in gram-negative bacteria, mediated by flavin-dependent monooxygenase variants, which was additionally excluded because YycH lacks the molecular basis of encoding enzymes and the determined tigecycline susceptibility in complemented strains co-expressing wild-type and Glu283Ter YycH. In summary, the resistant mechanism mediated by Glu283Ter YycH in TRSAms is different from tigecycline-resistant bacteria. Therefore, our hypothesis should be expanded to other antimicrobial resistance mechanisms in bacteria, including decreasing the uptake of drugs and metabolism redirection. Based on these two mechanisms, we propose two mechanistic hypotheses to explain tigecycline resistance, which is conferred if and only if Glu283Ter YycH is present: (i) Glu283Ter YycH may either directly hinder tigecycline intracellular permeation through the cytoplasmic membrane or have unknown biological functions that regulate other pathways to reduce the uptake of tigecycline. (ii) Tigecycline may interact with YycH to disrupt cell metabolism and potentiate its antibacterial effect, whereas the Glu283Ter mutation may decrease the binding affinity or redirect the metabolism pathway, thereby attenuating tigecycline effects.

Taken together, this study describes a comprehensive ALE under the selective pressure of tigecycline. As in the previous study, the *rpsJ* gene was initially detected with mutations associated with tigecycline resistance. Subsequently, the *mepA* genes conferred higher resistance against tigecycline through mutations and overexpression. Finally, the *yycH* gene was first demonstrated to be directly associated with tigecycline susceptibility.

To understand the potential clinical relevance of the above mutations in ALE, we scanned the global genomes. The results showed that the mutations on the *rpsJ* gene had been prevalent in *S. aureus* isolates. The mutation Asp60Tyr on the *rpsJ* gene, which is the same as this study, was discovered in an ALE mutant of *S. aureus* MS4 under omadacycline selective pressure (GCF_003992475.1). It demonstrates that mutant *rpsJ* conferred cross-resistance even between advanced tetracycline agents. Mutation Asp60Tyr was more common than Lys57Gln mutation and was detected in six countries across three continents. Notably, these isolates also carried various antibiotic-resistant genes to confer resistance against beta-lactams, aminoglycosides, bleomycin, and macrolides. Although the same mutations on *mepA* and *yycH* were not discovered, the clinical importance cannot be ignored. The mutant *mepA* has been reported in clinical cases (33), and given that a previous study (14) demonstrated that the MepA efflux pump could confer tigecycline resistance with mutations and overexpression, mutation detection of *mepA* should be considered as a molecular monitoring target when necessary. Moreover, mutant *yycH* has been implicated in resistance against various antimicrobial agents classified as “last-resort antibiotics” in *in vivo* studies (27, 28), and the *yycH* gene is widely distributed in low (G + C) percentage gram-positive bacteria, including *Bacillus*, *Listeria*, *Staphylococcus*, *Enterococcus*, and *Lactobacillus* spp. strains (25). It suggests that the YycH protein may be considered a potential mechanism of antimicrobial resistance and a candidate antibacterial target in gram-positive bacteria.

### Conclusion

The complete evolutionary trajectory of *S. aureus* in ALE under the selective pressure of tigecycline was depicted in the current study. Higher-level TRSAm strains were acquired from the selection of concentration gradients. A series of phenotypic assays were performed, demonstrating different characteristics in high-level TRSAms. Genomic analysis further detected widely novel synchronous mutations between *yycH* and *fakA*. Further cloning experiments, deletion mutant construction, and complementation confirmed that the *yycH* gene is involved in tigecycline susceptibility in *S. aureus*. Overall, this study presents a more comprehensive understanding of the tigecycline-resistant mechanism and a potential antimicrobial drug target in *S. aureus* and provides molecular reference through potential clinical relevance analysis. However, an unresolved issue in this study is the relationship between autolysis reduction and tigecycline treatment. The autolysis-related genes were not identified in the WGS analysis, which means that genomic changes cannot account for this phenomenon. Additionally, our results provide future perspectives on molecular mechanisms associated with the *yycH* gene, and further experiments will focus on determining how mutations in *yycH* contribute to decreased susceptibility to tigecycline. To address these issues, future research should focus on transcriptome analysis to identify the pathways and key genes involved in the ALE-driven decrease in tigecycline susceptibility.

## SUPPLEMENTAL MATERIAL

Figure S1 aac.00072-25-s0001.tif Gene expressions were determined by RT-qPCR.

Table S1 aac.00072-25-s0002.docx Primers used in the study.

Table S2 aac.00072-25-s0003.docx Completed results of antimicrobial susceptibility testing in the study.

Table S3 aac.00072-25-s0004.xlsx Genomic information of mutant *rpsJ*.

ASM does not own the copyrights to Supplemental Material that may be linked to, or accessed through, an article. The authors have granted ASM a non-exclusive, world-wide license to publish the Supplemental Material files. Please contact the corresponding author directly for reuse.
