# Supplementary figures and images for: Phenotypic and genetic stepwise changes in Staphylococcus aureus during in vitro adaptive laboratory evolution under the selective pressure of tigecycline
Source: Antimicrob Agents Chemother. 2025 Mar 26;69(5):e00072-25. doi: 10.1128/aac.00072-25 (PMC12057350; doi:10.1128/aac.00072-25)

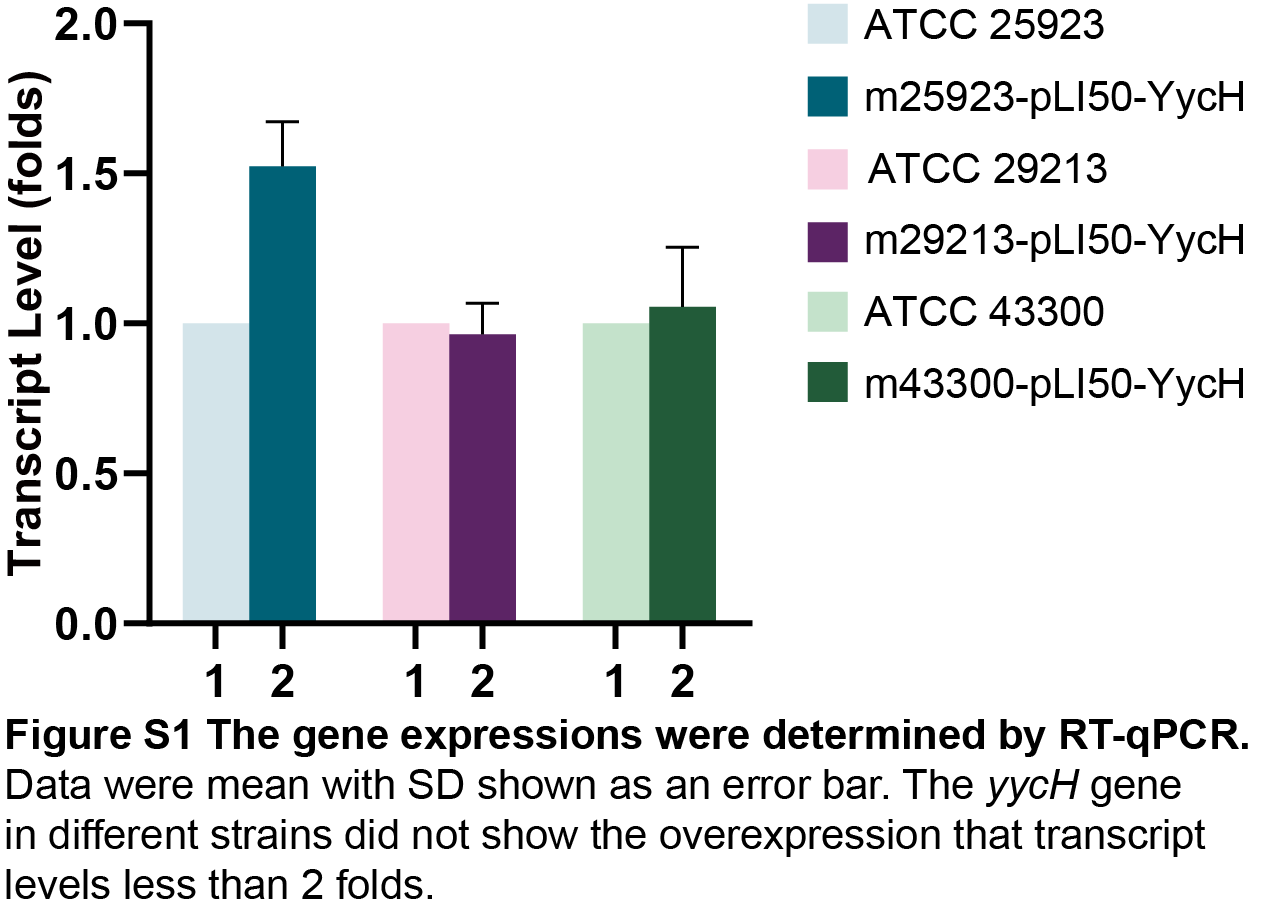

Supplement: Figure S1 — Gene expressions were determined by RT-qPCR. [file aac.00072-25-s0001.tif]
