# Supplementary material for: Phenotypic and genetic stepwise changes in Staphylococcus aureus during in vitro adaptive laboratory evolution under the selective pressure of tigecycline
Source: Antimicrob Agents Chemother. 2025 Mar 26;69(5):e00072-25. doi: 10.1128/aac.00072-25 (PMC12057350; doi:10.1128/aac.00072-25)
Supplement: Table S1 — Primers used in the study. [file aac.00072-25-s0002.docx]

**TABLE S1 Primers used in the study**

| **Names** | **Nucleotide sequences (5’-3’)** | **Descriptions** | **Sizes of production** |
| --- | --- | --- | --- |
| mepA-F | CACTCGTATCGCAGTTATCTG | Mutant detection on the *mepA* gene | 1703 bp |
| mepA-R | CTTTAACTTCTGATTCTTCACTA |  |  |
| rpsJ-F | GCCATAGAAAAACTCACAAAGT | Mutant detection on the *rpsJ* gene | 759 bp |
| rpsJ-R | GTCCACCTCCTAAAATTGTCT |  |  |
| yycH-F | AAATGGGTGGTACTGG | Mutant detection on the *yycH* gene | 1580 bp |
| yycH-R | TGTGAGCGATTGACTTTATT |  |  |
| fakA-F | GCTTAGGAGGACAACTTGAAAT | Mutant detection on the *fakA* gene | 1712 bp |
| fakA-R | CTACCAACTGATGATTTATCATTGGT |  |  |
| vLI-F | GCCCTCCCGTATCGTA | Verified the existence of plasmid pLI50 | 1042 bp |
| vLI-R | CGCTTCCTCGCTCACT |  |  |
| P1 | CGACTCTAGAGGATCTTCTCAGTTGGTCCAGATAGAGAAC | Construction of pLI-YycH | 1359 bp |
| P2 | CGATGGTGACGGTGATTGGGATGAATAATAAGGAACA |  |  |
| P3 | TCACCGTCACCATCGTATGCACAGTACACA |  | 1364 bp |
| P4 | TCGTCTTCAAGAATTTTCATTTATTCAAGCCTCCCATCGTTATAAACA |  |  |
| P5 | GGTACCCGGGAGCTCGAATTCGACCTGTTACGAAACATTG | Construction of pLI-FakA | 2522 bp |
| P6 | CCGGGTACCGAGCTCGAATTCTACCAACTGATGATTTATCATTGGT |  |  |
| P7 | GGTACCCGGGAGCTCGAATTGGTCTAATCGCACGTGTT | Construction of pHoss-hyycH | 893 bp |
| P8 | ATTTATTCAAGCCTCCCATCGACGAGTAGCGCTAAAATGACA |  |  |
| P9 | TGTCATTTTAGCGCTACTCGTCGATGGGAGGCTTGAATAAAT |  | 831 bp |
| P10 | CTGCAGAAGCTTCTAGAATTAGGGCTATTATTTGTCGCTTC |  |  |
