# Supplementary material for: Phenotypic and genetic stepwise changes in Staphylococcus aureus during in vitro adaptive laboratory evolution under the selective pressure of tigecycline
Source: Antimicrob Agents Chemother. 2025 Mar 26;69(5):e00072-25. doi: 10.1128/aac.00072-25 (PMC12057350; doi:10.1128/aac.00072-25)
Supplement: Table S2 — Completed results of antimicrobial susceptibility testing in the study. [file aac.00072-25-s0003.docx]

**Table S2 Completed results of antimicrobial susceptibility testing in the study.**

|  | OXA | PEN | CEF | FOX | GEN | AMI | TET | DOX | MNO | TGC | FFC |
| --- | --- | --- | --- | --- | --- | --- | --- | --- | --- | --- | --- |
| ATCC 43300 | 4 | 8 | 8 | 16 | 0.5 | 1 | 0.5 | 0.125 | 0.125 | 0.125 | 2 |
| m43300T2 | 0.5 | 4 | 2 | 4 | 1 | 1 | 2 | 0.5 | 0.5 | 2 | 2 |
| m43300T8 | 0.25 | 1 | 1 | 2 | 2 | 1 | 8 | 2 | 1 | 8 | 2 |
| m43300T32 | 0.25 | 0.25 | 1 | 2 | 2 | 1 | 16 | 4 | 4 | 32 | 2 |
| m43300T128 | 0.125 | 0.25 | 1 | 2 | 4 | 1 | 32 | 8 | 8 | 128 | 2 |
| ATCC 25923 | 1 | 1 | 2 | 2 | 1 | 1 | 0.5 | 0.125 | 0.125 | 0.125 | 4 |
| m25923T2 | 0.25 | 0.25 | 1 | 2 | 1 | 1 | 4 | 1 | 0.5 | 2 | 2 |
| m25923T8 | 0.25 | 0.25 | 2 | 2 | 2 | 1 | 8 | 2 | 2 | 8 | 2 |
| m25923T32 | 0.25 | 0.25 | 1 | 2 | 4 | 1 | 16 | 4 | 4 | 32 | 2 |
| m25923T128 | 0.25 | 0.25 | 1 | 2 | 4 | 2 | 32 | 16 | 8 | 128 | 2 |
| ATCC 29213 | 0.5 | 2 | 2 | 4 | 1 | 2 | 0.5 | 0.125 | 0.125 | 0.125 | 2 |
| m29213T2 | 0.25 | 1 | 1 | 4 | 1 | 2 | 2 | 0.25 | 0.25 | 2 | 2 |
| m29213T8 | 0.25 | 1 | 1 | 4 | 2 | 2 | 8 | 1 | 0.25 | 8 | 2 |
| m29213T32 | 0.125 | 0.25 | 1 | 2 | 4 | 2 | 16 | 4 | 2 | 16 | 2 |
| m29213T128 | 0.125 | 0.25 | 1 | 2 | 4 | 2 | 32 | 8 | 8 | 128 | 2 |

(Table S2 continue)

|  | ERY | TMI | RIF | VAN | CLI | TIA | CIP | ENR | LZD | TZD | SXT |
| --- | --- | --- | --- | --- | --- | --- | --- | --- | --- | --- | --- |
| ATCC 43300 | 0.5 | 1 | ≤0.004 | 1 | 1 | 1 | 0.5 | 0.25 | 1 | 0.125 | 2 |
| m43300T2 | 0.125 | 1 | ≤0.004 | 1 | 1 | 0.5 | 0.5 | 0.06 | 2 | 0.125 | 4 |
| m43300T8 | 0.125 | 1 | ≤0.004 | 1 | 1 | 0.5 | 0.5 | 0.06 | 2 | 0.125 | 4 |
| m43300T32 | 0.125 | 1 | ≤0.004 | 2 | 1 | 0.5 | 0.5 | 0.06 | 2 | 0.125 | 4 |
| m43300T128 | 0.125 | 1 | ≤0.004 | 2 | 1 | 0.5 | 0.5 | 0.06 | 1 | 0.125 | 2 |
| ATCC 25923 | 0.5 | 1 | ≤0.004 | 1 | 1 | 1 | 0.25 | 0.125 | 1 | 0.125 | 2 |
| m25923T2 | 0.25 | 1 | ≤0.004 | 2 | 0.5 | 0.5 | 1 | 0.06 | 2 | 0.125 | 8 |
| m25923T8 | 0.25 | 1 | ≤0.004 | 1 | 2 | 0.5 | 0.5 | 0.06 | 1 | 0.125 | 8 |
| m25923T32 | 0.25 | 1 | ≤0.004 | 2 | 1 | 0.5 | 1 | 0.06 | 2 | 0.125 | 8 |
| m25923T128 | 0.5 | 1 | ≤0.004 | 2 | 1 | 0.5 | 0.5 | 0.06 | 1 | 0.125 | 4 |
| ATCC 29213 | 0.5 | 1 | 0.0016 | 1 | 0.5 | 1 | 1 | 0.06 | 1 | 0.125 | 2 |
| m29213T2 | 0.5 | 1 | ≤0.004 | 1 | 1 | 0.5 | 0.125 | 0.125 | ≤0.5 | 0.125 | 8 |
| m29213T8 | 0.5 | 1 | ≤0.004 | 1 | 1 | 0.5 | 0.125 | 0.125 | ≤0.5 | 0.125 | 8 |
| m29213T32 | 0.5 | 1 | ≤0.004 | 2 | 0.25 | 0.5 | 0.5 | 0.06 | 1 | 0.125 | 4 |
| m29213T128 | 0.5 | 1 | ≤0.004 | 2 | 1 | 2 | 0.5 | 0.06 | 1 | 0.125 | 4 |

Abbreviation: OXA, oxacillin; PEN, penicillin; CEF, ceftiofur; FOX, cefoxitin; GEN, gentamicin; AMI, amikacin; TET, tetracycline; DOX, doxycycline; TGC, tigecycline; FFC, florfenicol; ERY, erythromycin; TMI, tilmicosin; RIF, rifampicin; VAN, vancomycin; CLI, clindamycin; TIA tiamulin; CIP, ciprofloxacin; ENR, enrofloxacin; LZD, linezolid; TZD, tedizolid; SXT, sulfamethoxazole-trimethoprim. Data presented with mg/L.
